# The influence of steroidal implants and manganese sulfate supplementation on growth performance, trace mineral status, hepatic gene expression, hepatic enzyme activity, and circulating metabolites in feedlot steers

**DOI:** 10.1093/jas/skae062

**Published:** 2024-03-08

**Authors:** Dathan T Smerchek, Emma L Rients, Amy M McLaughlin, Jacob A Henderson, Brock M Ortner, Kara J Thornton, Stephanie L Hansen

**Affiliations:** Department of Animal Science, Iowa State University, Ames, IA, 50011, USA; Department of Animal Science, Iowa State University, Ames, IA, 50011, USA; Department of Animal Science, Iowa State University, Ames, IA, 50011, USA; Department of Animal Science, Iowa State University, Ames, IA, 50011, USA; Department of Animal Science, Iowa State University, Ames, IA, 50011, USA; Department of Animal, Dairy, and Veterinary Science, Utah State University, Logan, UT, 84322, USA; Department of Animal Science, Iowa State University, Ames, IA, 50011, USA

**Keywords:** cattle, feedlot, implant, manganese

## Abstract

Angus-cross steers (*n* = 144; 359 kg ± 13.4) were used to assess the effect of dietary Mn and steroidal implants on performance, trace minerals (TM) status, hepatic enzyme activity, hepatic gene expression, and serum metabolites. Steers (*n* = 6/pen) were stratified by BW in a 3 × 2 factorial. GrowSafe bunks recorded individual feed intake (experimental unit = steer; *n* = 24/treatment). Dietary treatments included (MANG; 8 pens/treatment; Mn as MnSO_4_): (1) no supplemental Mn (analyzed 14 mg Mn/kg DM; Mn0); (2) 20 mg supplemental Mn/kg DM (Mn20); (3) 50 mg supplemental Mn/kg DM (Mn50). Within MANG, steers received a steroidal implant treatment (IMP) on day 0: (1) no implant; NO; or (2) combination implant (Revalor-200; REV). Liver biopsies for TM analysis and qPCR, and blood for serum glucose, insulin, non-esterified fatty acids, and urea-N (SUN) analysis were collected on days 0, 20, 40, and 77. Data were analyzed as a randomized complete block with a factorial arrangement of treatments including fixed effects of Mn treatment (MANG) and implant (IMP) using PROC MIXED of SAS 9.4 using initial BW as a covariate. Liver TM, serum metabolite, enzyme activity, and gene expression data were analyzed as repeated measures. No MANG × IMP effects were noted (*P *≥ 0.12) for growth performance or carcass characteristic measures. Dietary Mn did not influence final body weight, overall ADG, or overall G:F (*P* ≥ 0.14). Liver Mn concentration increased with supplemental Mn concentration (MANG; *P *= 0.01). An IMP × DAY effect was noted for liver Mn (*P *= 0.01) where NO and REV were similar on day 0 but NO cattle increased liver Mn from days 0 to 20 while REV liver Mn decreased. Relative expression of MnSOD in the liver was greater in REV (*P *= 0.02) compared to NO and within a MANG × IMP effect (*P *= 0.01) REV increased liver MnSOD activity. These data indicate current NASEM Mn recommendations are adequate to meet the demands of finishing beef cattle given a steroidal implant. Despite the roles of Mn in metabolic pathways and antioxidant defense, a basal diet containing 14 mg Mn/kg DM was sufficient for the normal growth of finishing steers. This study also provided novel insight into how implants and supplemental Mn influence genes related to arginine metabolism, urea synthesis, antioxidant capacity, and TM homeostasis as well as arginase and MnSOD activity in hepatic tissue of beef steers.

## Teaser

Supplementing Mn at the current recommended concentrations does not restrict growth of either implanted or non-implanted cattle. Additionally, our data indicate liver Mn correlates with liver arginase activity and circulating serum urea nitrogen, suggesting liver Mn concentration is more responsive to alterations in nitrogen metabolism than dietary Mn.

## Introduction

Manganese (Mn) is an essential trace element, which serves as a cofactor and activator of several enzymatic complexes. The Mn requirement for growing cattle is 20 mg of Mn/kg of dry matter (DM; NASEM, 2016). However, a survey of consulting nutritionists conducted by Samuelson et al. (2016) reports Mn, amongst other trace minerals (TM), is commonly supplemented at concentrations exceeding NASEM (2016) recommendations (50 mg Mn/kg DM). Limited work has investigated solely supplemental Mn concentration in finishing cattle (Legleiter et al., 2005).

The liver contributes to whole-body Mn homeostasis via flexible Mn excretion (Miller, 1973; Hambidge et al., 1989). Liver Mn absorption, excretion, and reuptake appear to be under strict homeostatic control by the metal transporters ZIP8, ZIP14, and ZnT10 in other species (Tuschl et al., 2012, 2016; Boycott et al., 2015). Interestingly, steroidal implant administration routinely decreases liver Mn concentrations in finishing cattle (Niedermayer et al., 2018; Messersmith et al., 2021; Reichhardt et al., 2021). Manganese supports nitrogen recycling in the ruminant animals through its role in the urea cycle, as a factor in the Mn-dependent enzyme arginase (Lapierre and Lobley, 2001) and as a primary mitochondrial antioxidant defense in MnSOD. However, the effects of supplemental Mn concentration in implanted vs. non-implanted finishing cattle have not been explored.

The study objective was to determine the effects of increasing Mn supplementation on growth performance, carcass characteristics, circulating metabolites, hepatic expression of genes related to arginine metabolism, urea synthesis, antioxidant capacity, and TM homeostasis and activity of hepatic arginase and MnSOD, as well as markers of TM metabolism in implanted vs. non-implanted finishing beef steers. We hypothesized that increasing Mn supplementation would alter Mn-dependent hepatic enzyme activity related to urea synthesis, antioxidant capacity, and markers of TM metabolism to support increased steroidal implant-induced growth.

## Materials and Methods

All procedures and protocols were approved by the Iowa State University Institutional Animal Care and Use Committee (IACUC-20-127).

### Experimental design

Angus-cross steers (*n* = 144; 359 kg ± 13.38) from a single ranch (Valentine, NE) were utilized in a study conducted at the beef nutrition farm located in Ames, IA from mid-November 2022 to late April 2023. Prior to this finishing trial, calves were enrolled in a 28 d receiving trial with varying dietary potassium treatments, and were implanted with Revalor-IS (Merck Animal Health, Madison, NJ) 80 d prior to terminal implant. Previous receiving trial treatments were accounted for during allotment to the present study. To accommodate sampling logistics steers were assigned to two blocks (*n* = 72 steers/block; *n* = 12 pens/block). Blocks started on trial with a 14-d stagger with identical sampling and experimental procedures. Pens were equipped with GrowSafe^®^ feed bunks (*n* = 1 GrowSafe feed bunk/pen of 6 steers; GrowSafe^®^ Systems Ltd., Airdire, AB, Canada). Individual radio frequency tags in the ear of an individual steer allowed for recognition of feed disappearance associated with the corresponding individual radio frequency tags. These data are relayed from the bunk to GrowSafe software. Thus, individual intake data were recorded for each animal in each pen.

Steers were stratified by BW into a 3 × 2 factorial design taking into account receiving trial dietary treatment. Dietary treatments (MANG; 8 pens/treatment; Mn as MnSO_4_) included: (1) no supplemental Mn (analyzed 14 mg Mn/kg DM; Mn0); (2) 20 mg supplemental Mn/kg DM (analyzed 33 mg Mn/kg DM; Mn20); (3) 50 mg supplemental Mn/kg DM (analyzed 57 mg Mn/kg DM; Mn50). Dietary treatments began 55 d prior to implant and continued until cattle were harvested. This study focused on the terminal implant window, the last ~90 d prior to harvest. Thus, dietary Mn treatments began prior to terminal implant delivery, as cattle would receive TM supplementation during the growing phase prior to terminal implant. Within MANG treatment, steers received a steroidal implant treatment on day 0: (1) no implant; NO; or (2) high potency combination implant (Revalor-200, Merck Animal Health, Madison, NJ); (200 mg TBA + 20 mg E_2_; REV). Recent data indicates administration of an uncoated terminal implant 85-100 days prior to harvest is optimal for growth performance (Coulson et al., 2019). Cattle began dietary MANG treatments on day −55. Thus, the period from days −55 to −1 represents the window of Mn supplementation leading up to implant.

The day of implant was considered day 0, and sampling days are discussed and represented relative to implant administration (day 0). Individual steer BW were recorded at study initiation (days −56 and −55) prior to placement in final study pen as well as days −1, 0 (day of implant), 29, 56, 88, and 89. On day 90 steers were harvested at a commercial abattoir (National Beef, Tama, IA) via industry-accepted practices. Trained personnel collected hot carcass weight (HCW) data on the day of harvest while REA, 12th rib fat (RF), USDA yield grade, KPH, and marbling data were collected after a 48-h chill. Steer was the experimental unit and *n* = 24 per treatment mean for all performance variables.

### Dietary management

Cattle were fed a roughage-based diet from days −55 to −31, transitioned to a high concentrate diet, and on finishing diet for 24 d before implant on day 0. Cattle were fed treatment diets (Table 1) once daily at approximately 0800 hours. Steers were fed ad libitum during the entirety of the study with bunks managed to ensure residual feed remained in GrowSafe bunks between daily feed deliveries. Dietary treatments were included in the total mixed ration (TMR) as a premix with dried distiller grains plus solubles as a carrier. Water was provided ad libitum throughout the study via automatic waterers available in each pen. Water tanks were checked daily and cleaned by feedlot personnel as needed to ensure a constant and clean water supply to the cattle.

**Table 1.** Diet composition^1^

% DM basis			
	Transition 1	Transition 2	Finisher
Days fed	−55 to −41	−40 to –25	−24 to harvest
Ingredient			
Dry-rolled corn	20	30	50
Sweet Bran	30	27	20
Corn silage	30	23	15
DDGS	10	10	5
Mn premix^2^	5	5	5
Basal premix^3^	5	5	5
Formulated composition, %			
Crude protein^4^	16.3	15.9	13.8
NDF^4^	26.9	24.1	19.2
Ether extract^4^	4.4	4.5	4.4
NEm, Mcal/kg^4^	1.97	2.01	2.06
NEg, Mcal/kg^4^	1.33	1.36	1.39

^1^Provided vitamins at 2016 NASEM recommendations. The control diet analyzed 14 mg Cu/kg DM, 63 mg Fe/kg DM, 14 mg Mn/kg DM, and 66 mg Zn/kg DM.

^2^Treatment premix and basal utilized DDGS as a carrier and replaced DDGS in the diet. Dietary treatments included (1) no supplemental Mn (analyzed 14 mg Mn/kg DM; Mn0); (2) 20 mg supplemental Mn/kg DM (Mn20); (3) 50 mg supplemental Mn/kg DM (Mn50).

^3^Basal provided as % DM; limestone (1.5%), Rumensin (0.0135%), and salt (0.31%). Trace minerals and vitamins provided per kg of DM: 0.15 mg Co (cobalt carbonate), 20 mg Cu (copper sulfate), 0.1 mg Se (sodium selenite), 0.5 mg I (calcium iodate), 30 mg Zn (Zn sulfate), and Vitamin A 2,200 IU.

^4^Calculated from tabular energy values.

### Sample collection and analytical procedures

Samples of TMR were collected weekly. Samples were dried in a forced-air oven at 70 °C for 48 h for determination of DM percentage. Individual steer DM intake (DMI) was calculated from as-fed intakes (feed disappearance tracked by the unique EID assigned to each steer prior to study initiation) corrected for the DM (%) of weekly TMR samples. Samples of the control treatment TMR were dried, ground, and composited for analysis of nitrogen, neutral detergent fiber, and ether extract by a commercial laboratory (Dairyland Laboratories, Inc., Arcadia, WI). Gain:feed (G:F) was calculated from the total gain and total DMI during weighing intervals. Dried, ground, and composited TMR were acid digested using trace mineral grade nitric acid as previously described (Genther-Schroeder et al., 2016) before analysis for Cu, Fe, Mn, and Zn concentrations using inductively coupled plasma optical emission spectroscopy (Optima 7,000; PerkinElmer, Waltham, MA).

Liver biopsies were collected from all steers (*n* = 144) on days 0, 20, 40, and 77 relative to implant administration using the methods outlined by Engle and Spears (2000) and a sub-sample was frozen at −20 °C for TM analysis and −80 °C for enzyme activity analysis. Liver samples from all steers (*n* = 144; 24 steers/treatment) were used for analysis of liver TM concentration. Liver samples were dried and acid digested in preparation for subsequent analysis of Cu, Fe, Mn, and Zn concentration via ICP-OES using methods previously described (Pogge and Hansen, 2013). A bovine liver reference sample from National Institutes of Standards and Technology (Gaithersburg, MD) was included in all analyses to verify instrument accuracy.

Jugular blood samples were collected from all steers (*n* = 144) on days −55, 0, 20, 40, and 77 at least 2 h post-feeding immediately prior to liver and muscle biopsies. Whole blood samples (collected in no additive tubes for serum) were centrifuged at 1,000 × *g* for 20 min at 4 °C. Serum was aliquoted and stored at −80 °C prior to sample analysis.

A subset of steers (*n* = 72; 12 steers/treatment) were selected for analysis of circulating metabolites. Serum glucose, insulin, non-esterified fatty acids (NEFA), and urea-N were determined on days 0, 20, 40, and 77 relative to implant using commercially available kits; glucose (FUJIFILM Wako Diagnostics; intra-assay CV = 7.8%, inter-assay CV = 6.6%), insulin (Bovine Insulin ELISA assay; Mercodia, Inc., Winston Salem, NC; intra-assay CV = 4.1%, inter-assay CV = 4.4%), NEFA (Wako Pure Chemical Industries Ltd., Chuo-Ku Osaka, Japan; intra-assay CV = 3.2%, inter-assay CV = 5.6%), SUN (Teco Diagnostics, Anaheim, CA; intra-assay CV = 2.9%, inter-assay CV = 7.7%). Absorbances for plate-based assays were measured using a BioTek Eon (Agilent Technologies, Winooski, VT). Glucose, NEFA, and insulin concentrations were used to calculate revised quantitative insulin sensitivity check index (RQUICKI) which was calculated using an equation from de Sousa et al. (2022): RQUICKI = 1/[log(Glucose) + log(Insulin) + log(NEFA)].

Liver arginase activity (*n* = 72 steers; 12/treatment) was assessed in duplicate according to methods described by Messersmith et al. (2022). Approximately 50 mg of liver tissue (wet basis) was used for measurement of arginase activity. Sample urea production was normalized to protein content of the sample analyzed using a commercial Coomassie Bradford Protein Assay kit (Thermo Fisher Scientific, Waltham, MA) and arginase activity was determined by the amount of urea produced per µg protein in 1 min (nM urea/µg protein/min). Intra-assay and inter-assay CV for the protein-normalized arginase activity were 9.8% and 10.6%, respectively.

Liver MnSOD activity was determined (*n* = 72 steers; 12/ treatment) in duplicate using a commercially available kit (#706002, Cayman Chemical). Liver tissue (50 mg tissue; wet basis) was homogenized in 0.50 mL of 20 mM HEPES buffer, centrifuged at 1,500 × *g* for 5 min at 4 °C, and the supernatant was removed, aliquoted, and stored at − 80 °C until further analysis (#706002, Cayman Chemical). Inter- and intra-assay CV for MnSOD activity were 9.5% and 11.5%, respectively. Protein concentration was determined using a commercially available kit (#23200, Thermo Scientific, Rockford, IL) and liver MnSOD activity is reported as U/mg protein.

Liver samples from eight steers per treatment were utilized to analyze the expression of genes related to arginine metabolism, urea synthesis, antioxidant capacity, TM transporters, and enzymes and factors related to antioxidant capacity at all four sampling times (0, 20, 40, and 77). Liver samples were ground using a mortar and pestle in liquid nitrogen. Isolation of RNA and cDNA synthesis were conducted as described by (Rients et al., 2023). The cDNA product was stored at −20 °C until quantitative real-time polymerase chain reaction (qPCR) analysis. The quantity and quality of RNA were measured on a Cytation5 (Agilent Technologies, Winooski, VT) with a Take3Trio (Agilent Technologies) using a nucleic acid quantification protocol. Quantitative real-time PCR was performed as described by McGill et al. (2016) using ribosomal protein S9 (RPS9) as a reference gene (Janovick-Guretzky et al., 2007; Ostrowska et al., 2014). Amplification conditions for all genes were the same: (Hold stage) 2 min at 50 °C, 10 min at 95 °C, 40 cycles of 15 s 95 °C, and 1 min 60 °C (measure fluorescence step) and a dissociation step of 15 s 95 °C, 1 min 60 °C, 15 s 95 °C, and 15 s 60 °C. Dissociation curves were analyzed for proper product amplification. Primers utilized for qPCR can be found in Supplementary Table S1. Relative gene expression was determined by using the 2−ΔΔCt method (Livak and Schmittgen, 2001) and calculations were conducted relative to Mn0-NO treatment on day 0. Relative gene expression data are presented in Supplementary Table S2.

Supplementary Tables S3 and S4) show a series of weak, yet significant, correlations amongst liver Mn, serum urea nitrogen, and liver arginase activity that perhaps hint at an influence of liver urea synthesis demands on liver Mn concentrations. While not further discussed herein, it would be beneficial to further explore this relationship to provide clarity on the value of liver Mn concentrations in veterinary diagnostic situations.

### Statistical analysis

Feedlot growth and carcass data were analyzed as a randomized complete block design using the MIXED procedure of SAS 9.4 (SAS Inst. Inc., Cary, NC). Data were analyzed as a factorial including fixed effects of Mn treatment (MANG) and implant (IMP). Liver TM data, serum metabolite data, enzyme activity data, and gene expression data were analyzed as repeated measures using the MIXED procedure of SAS 9.4 (SAS Inst. Inc.). Individual steer was the experimental unit. The model included the fixed effects of MANG and IMP and the interaction with day as the repeated effect. The Correlation Procedure of SAS was utilized to assess correlations between liver arginase activity, SUN concentrations, and liver Mn concentrations. Initial BW (day −55) served as a covariate in growth performance data analysis. Including initial BW in the model as a covariate reduced variance in outcome variables. As no liver sample was collected on day −55 when dietary treatments began, no covariate was included for these measures during subsequent data analysis. Statistical outliers were determined as data beyond three standard deviations from the mean for a particular parameter and were removed. Significance was determined as *P* ≤ 0.05 and tendencies were declared when 0.05 < *P* ≤ 0.10.

## Results

### Growth performance and carcass characteristics

#### Pre-implant period performance (days −55 to 0)

Bodyweight at study initiation (day −55) was 360 ± 13 kg and did not differ (*P *≥ 0.42) between MANG treatments (Table 2). Average daily gain, DMI, and G:F were not influenced by MANG (*P *≥ 0.26). Following 55 d of dietary treatments, body weight at the time of implant administration (day 0) did not differ (*P *≥ 0.22) between treatments.

**Table 2.** Mn supplementation and steroidal implants influence on live growth performance in beef steers^1^

		MANG^2^			IMP^3^				*P*-value	
	Mn0	Mn20	Mn50	SEM	NO	REV	SEM	MANG	IMP	MANG × IMP
*Day −55 to 0*										
day −55 BW, kg	359	362	358	1.9	360	360	1.6	0.42	0.99	0.21
ADG, kg/d	1.87	1.95	1.85	0.043	1.89	1.89	0.035	0.26	0.91	0.37
DMI, kg/d	9.9	10.1	9.9	0.14	10.0	10.0	0.11	0.52	0.97	0.78
G:F	0.190	0.192	0.188	0.0036	0.189	0.190	0.0030	0.70	0.76	0.12
*Day 0 to 56*										
day 0 BW, kg	463	467	461	2.3	464	464	1.9	0.22	0.96	0.34
day 56 BW, kg	561	560	565	2.9	556	569	2.4	0.49	0.01	0.66
ADG, kg/d	1.76^ab^	1.68^b^	1.82^a^	0.036	1.65	1.86	0.029	0.02	0.01	0.35
DMI, kg/d	10.6	10.6	10.8	0.17	10.6	10.7	0.13	0.69	0.77	0.78
G:F	0.168^x^	0.159^y^	0.168^x^	0.0034	0.155	0.174	0.0027	0.08	0.01	0.25
*Day 56 to 89*										
Final BW	617	613	612	3.7	604	624	3.0	0.63	0.01	0.55
ADG, kg/d	1.66^a^	1.59^ab^	1.48^b^	0.055	1.44	1.71	0.045	0.06	0.01	0.43
DMI, kg/d	11.1	11.1	11.2	0.17	11.1	11.2	0.14	0.96	0.33	0.57
G:F	0.143^a^	0.137^ab^	0.129^b^	0.0044	0.127	0.146	0.0035	0.10	0.01	0.49
*Overall*										
Overall ADG, kg/d	1.74	1.66	1.69	0.028	1.58	1.82	0.023	0.14	0.01	0.22
Overall DMI, kg/d	10.9	10.9	11.0	0.15	10.9	11.0	0.13	0.89	0.34	0.77
Overall G:F	0.157	0.150	0.153	0.0026	0.145	0.163	0.0021	0.18	0.01	0.25

^1^Day −55 BW served as a covariate in analysis for all growth performance measures except for day −55 BW.

^2^Dietary treatments supplemented as MnSO_4_: (1) no supplemental Mn (analyzed 14 mg Mn/kg DM; Mn0); (2) 20 mg supplemental Mn/kg DM (Mn20); (3) 50 mg supplemental Mn/kg DM (Mn50).

^3^A Revalor-200 implant was administered to REV on day 0 (Merck Animal Health, Madison, NJ); 200 mg TBA + 20 mg E2; REV).

^a,b^Within rows, means without a common superscript differ (*P* ≤ 0.05).

^x,y^Within rows, means without a common superscript differ (0.05 < *P* ≤ 0.10).

#### Implant period performance

DMI (overall, or interim period) was not affected by MANG, IMP, or MANG × IMP (*P *≥ 0.33). Overall (days 0 to 89), final body weight, ADG, and G:F were not influenced by MANG × IMP (*P *≥ 0.22), or MANG (*P *≥ 0.14). Steroidal implant administration resulted in greater final body weight, and overall ADG and G:F (*P *≤ 0.01). Within interim periods, dietary Mn treatment influenced days 0 to 56 ADG (*P *= 0.02) where Mn50 was greater than Mn20 with Mn0 intermediate. Additionally, days 0 to 56 G:F tended to be influenced by MANG (*P *= 0.08) where Mn0 and Mn50 were greater than Mn20. From days 56 to 89 Mn0 tended to have greater ADG and G:F (*P *≤ 0.10) than Mn50 cattle with Mn20 being intermediate.

No MANG × IMP effects were noted (*P *≥ 0.12) for any carcass characteristics (Table 3). Steroidal implant resulted in greater HCW (*P *= 0.01) and tended to increase REA (*P *= 0.07). Additionally, REA was greater in Mn0 than Mn20, with Mn50 intermediate (MANG; *P = *0.01). Steroidal implant administration did not affect RF (*P *= 0.82), but MANG treatment did tend to influence RF (*P *= 0.08), where Mn50 had greater RF than Mn0 or Mn20. Calculated yield grade, MS, KPH, and dressing percentage were not influenced by MANG or IMP (*P *≥ 0.25).

**Table 3.** Mn supplementation and steroidal implants influence on carcass characteristics in beef steers^1^

		MANG^3^				IMP^4^			*P*-Value	
	Mn0	Mn20	Mn50	SEM	NO	REV	SEM	MANG	IMP	MANG × IMP
HCW, kg	393	391	390	2.0	384	398	2.0	0.54	0.01	0.20
REA, cm^2^	84.6^a^	81.3^b^	82.6^ab^	0.8	82.0	83.7	0.6	0.01	0.07	0.98
RF, cm	1.53^b^	1.50^b^	1.68^a^	0.06	1.56	1.58	0.05	0.08	0.82	0.26
DP, %	63.9	63.6	63.7	0.002	63.6	63.8	0.002	0.55	0.35	0.45
Yield Grade^5^	3.56	3.64	3.91	0.086	3.65	3.83	0.069	0.25	0.84	0.23
Marbling^2^	489	514	493	12.4	483	495	10.2	0.45	0.34	0.94
KPH, %	2.7	2.6	2.6	0.11	2.2	2.8	0.09	0.36	0.34	0.12

^1^Day −55 BW served as a covariate in analysis. Hot carcass weight (HCW), ribeye area (REA), 12th rib fat (RF), dressing percentage (DP), empty body fat percentage (EBF), kidney, pelvic, and heart fat (KPH).

^2^Marbling scores: slight: 300, small: 400, and modest: 500.

^3^Dietary treatments supplemented as MnSO_4_: (1) no supplemental Mn (analyzed 14 mg Mn/kg DM; Mn0); (2) 20 mg supplemental Mn/kg DM (Mn20); (3) 50 mg supplemental Mn/kg DM (Mn50).

^4^A Revalor-200 implant was administered to REV on day 0 (Merck Animal Health, Madison, NJ); 200 mg TBA + 20 mg E2; REV).

^5^Yield grade (YG) was calculated utilizing the USDA yield grade equation.

^a,b^Within rows, means without a common superscript differ (*P* ≤ 0.05).

^x,y^Within rows, means without a common superscript differ (0.05 < *P* ≤ 0.10).

#### Liver TM

**Figure 1.**
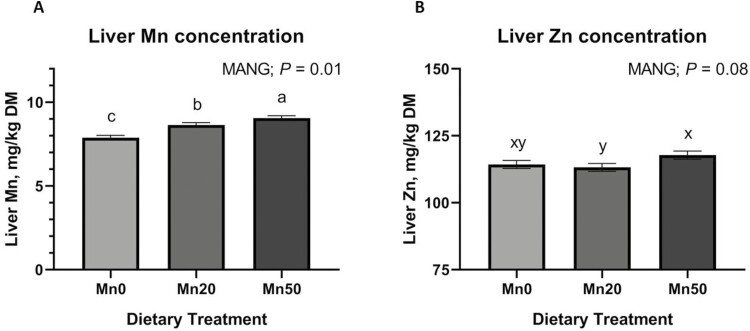
The effect of MANG on liver Mn and liver Zn concentration. Data were analyzed as repeated measures of the mixed procedure of SAS. Within a panel, ^a,b^unlike superscripts differ (*P* ≤ 0.05); ^x,y^unlike superscripts tend to differ (0.05 < *P* ≤ 0.10).

**Figure 2.**
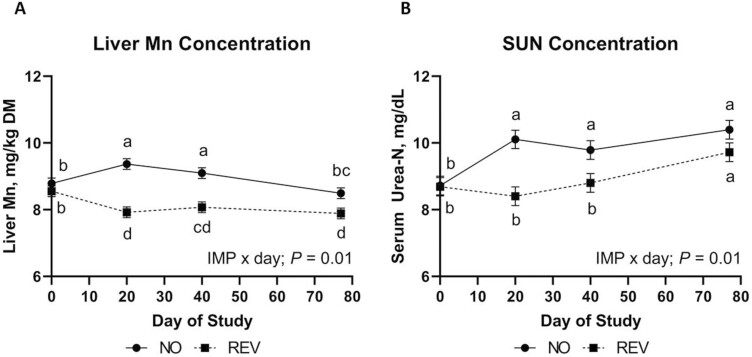
The effect of implant treatment across DOF (IMP × day) on liver Mn concentration and serum urea-N (SUN). Data were analyzed as repeated measures of the mixed procedure of SAS. Within a panel, ^a,b^unlike superscripts differ (*P* ≤ 0.05).

Liver TM concentrations were not influenced by MANG × IMP or MANG × IMP × DAY (data not shown; *P *≥ 0.16). Liver Mn concentration (Figure 1A) was influenced by MANG (*P *= 0.01) where Mn50 had the greatest overall liver Mn, Mn0 the least, with Mn20 being intermediate. An IMP × DAY effect (Figure 2A) was noted for liver Mn (*P *= 0.01) where NO and REV were not different on d 0 but NO cattle increased liver Mn from days 0 to 20 while REV liver Mn values decreased during this time. The difference in liver Mn between IMP treatments persisted for the remainder of the study even though liver Mn in NO decreased from days 40 to 77. Liver Fe concentration was not influenced by IMP or IMP × DAY (*P *≥ 0.41). A MANG × DAY effect was observed (*P *= 0.01) where liver Fe concentrations were not different on days 0, 20, and 77 but on day 40, Mn20 had greater liver Fe compared to Mn0 and Mn50. Liver Zn concentration was not influenced by IMP, IMP × DAY, or MANG × DAY (*P *≥ 0.34). Liver Zn concentration (Figure 1B) tended to be affected by MANG treatment (*P *= 0.08) where Mn50 tended to have the greatest liver Zn concentration, Mn0 was intermediate, and Mn20 was least. Liver Zn increased from days 0 to 20 and decreased from day 20 through day 77 (DAY; *P *= 0.01) where concentrations were 115.0, 119.9, 114.7, and 110.8 (SEM = 1.74) mg Zn/kg DM on days 0, 20, 40, and 84, respectively. Liver Cu concentration was not affected by MANG, IMP, MANG × DAY, or IMP × DAY (*P *≥ 0.31). However, liver Cu concentration increased from days 0 to 20, maintained to day 40 and decreased from days 40 to 77 (DAY; *P* = 0.01) where concentrations were 279.7, 294.9, 289.3, and 274.8 (SEM = 6.29) mg Cu/kg DM on days 0, 20, 40, and 84, respectively.

### Serum metabolites

**Figure 3.**
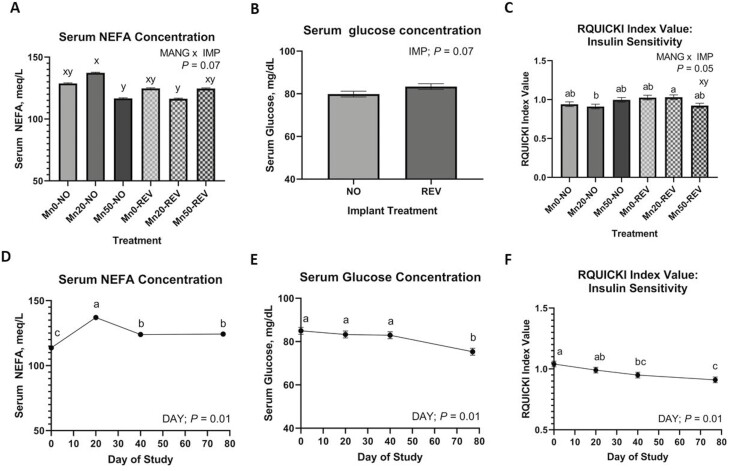
The effects of MANG × IMP and DAY on serum non-esterified fatty acids, the effects of IMP and DAY on serum glucose, and the effects of MANG × IMP and DAY on revised quantitative insulin sensitivity check index (RQUICKI) index value. Data were analyzed as repeated measures of the mixed procedure of SAS. Within a panel, ^a,b^unlike superscripts differ (*P* ≤ 0.05); ^x,y^unlike superscripts tend to differ (0.05 < *P* ≤ 0.10).

**Figure 4.**
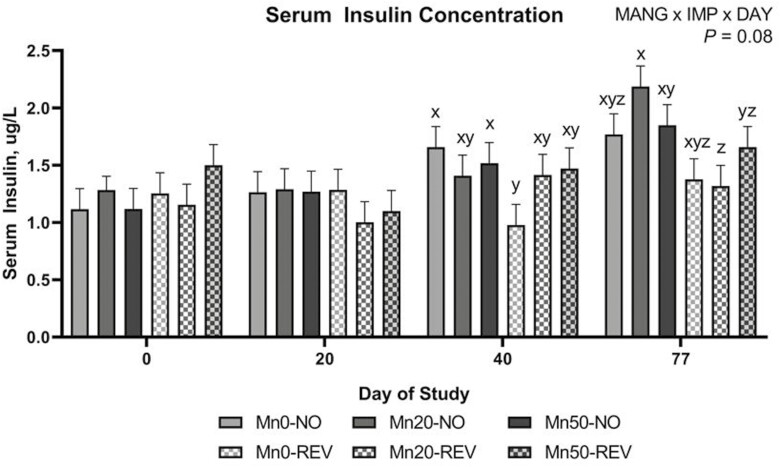
The effect of implant treatment across DOF (MANG × IMP × DAY) on serum insulin concentration. Data were analyzed as repeated measures of the mixed procedure of SAS. Within day, a,bunlike superscripts differ (*P* ≤ 0.05).

Serum urea-N was not influenced by MANG, MANG × DAY, MANG × IMP, or MANG × IMP × DAY (*P *≥ 0.25). An IMP × DAY effect (Figure 2B) was noted for circulating SUN where NO and REV were not different on day 0, NO increased to day 20 and remained greater through day 40 while REV remained the same through day 40. This difference persisted until day 77 where NO and REV were not different. Serum glucose was not affected by MANG, MANG × DAY, MANG × IMP, or MANG × IMP × DAY (*P *≥ 0.11) but tended to be greater (IMP; *P *= 0.07) in REV compared to NO (Figure 3B). Serum glucose was not different on days 0, 20, and 40 decreasing to its lowest point by day 77 (Figure 3E; DAY; *P *= 0.01). A tendency for a MANG × IMP × DAY effect was noted (*P *= 0.08) for serum insulin (Figure 4) which was driven partially by Mn0-REV decreasing from days 20 to 40 while all other treatments increased or remained similar. Serum NEFA was not affected by MANG, IMP, MANG × DAY, or MANG × IMP × DAY (*P *≥ 0.26). A MANG × IMP tendency was noted (*P *= 0.07) for serum NEFA concentration (Figure 3A) where Mn20-NO was greatest, Mn20-REV and Mn50-NO were lesser, with Mn0-REV, Mn50-REV, and Mn0-NO intermediate. Serum NEFA changed over time (Figure 3D; DAY; *P *= 0.01) where NEFA concentrations were lowest on day 0, greatest on day 20, and intermediate on days 40 and 77. Serum RQUICKI index value was not affected by MANG, IMP, MANG × DAY, IMP × DAY, or MANG × IMP × DAY (*P *≥ 0.14). A MANG × IMP effect was noted for RQUICKI (Figure 3C; *P *= 0.05) where Mn20-REV was greatest, Mn20-NO was least, with remaining treatments intermediate. The calculated RQUICKI value decreased over time (Figure 3F; DAY; *P *= 0.02).

### Liver tissue relative gene expression and hepatic enzyme activity

Relative gene expression of enzymes related to urea synthesis, Mn transporters, and enzymes and factors related to antioxidant capacity were assessed in crude liver homogenate of steers prior to implant (day 0) and on days 20, 40, and 77 relative to implant administration. Relative gene expression data are presented in Supplementary Table S2.

#### Arginine metabolism/urea synthesis

**Figure 5.**
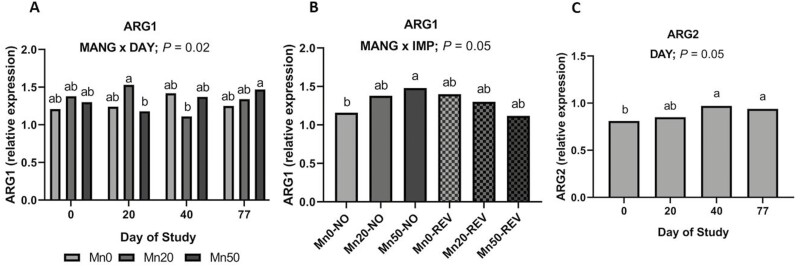
The effects of MANG × DAY and MANG × IMP on hepatic arginase 1 (ARG1) relative expression and the effect of DAY on arginase 2 (ARG2) relative expression. Data were analyzed as repeated measures of the mixed procedure of SAS. Within a panel, ^a,b^unlike superscripts differ (*P* ≤ 0.05); ^x,y^unlike superscripts tend to differ (0.05 < *P* ≤ 0.10).

A MANG × DAY effect was noted (Figure 5A; *P *= 0.01) for the cytosolic isoform of the Mn-dependent enzyme arginase (ARG1), driven by increased relative expression of ARG1 in Mn20 on day 20. Additionally, a MANG × IMP effect was noted (Figure 5B; *P *= 0.05) driven by differences within NO while implanted cattle had similar relative expression of ARG1. Relative expression of the mitochondrial isoform of arginase (ARG2) changed over time (Figure 5C; DAY; *P *= 0.05) where expression was lowest on day 0, increased to day 40, and remained similar through day 77. Other genes associated with arginine metabolism and urea synthesis, nitric oxide synthase 2 (NOS2), and carbamoyl-phosphate synthetase 1, were not influenced by the main effects of IMP, MANG, and DAY or their interactions (*P *≥ 0.14).

#### Antioxidant capacity

**Figure 6.**
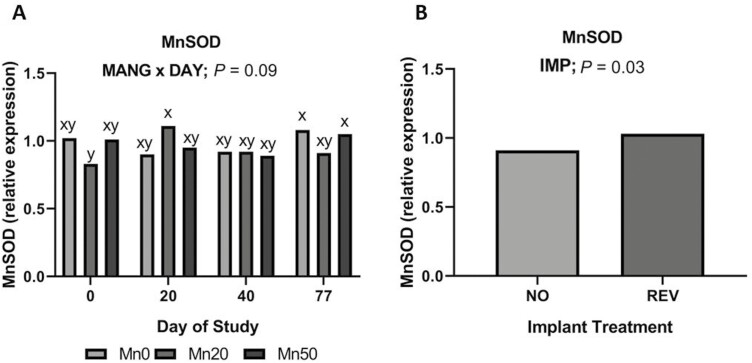
The effect of MANG × DAY and IMP on hepatic relative expression of MnSOD. Data were analyzed as repeated measures of the mixed procedure of SAS. Within a panel, ^a,b^unlike superscripts differ (*P* ≤ 0.05); ^x,y^unlike superscripts tend to differ (0.05 < *P* ≤ 0.10).

Relative expression of Mn superoxide dismutase (MnSOD) was greater in REV (Figure 6B; *P *= 0.02) compared to NO. Additionally, a tendency for a MANG × DAY effect (Figure 6A) was noted (*P *= 0.09) for MnSOD expression that was driven by Mn20 tending to have the lowest MnSOD expression on days 0 and 77 and greatest expression on day 20 compared to Mn0 and Mn50. Nuclear factor erythroid 2 (NRF2) expression was not influenced by the main effects of IMP, MANG, and DAY or their interactions (*P *≥ 0.29).

#### Trace metal transporters

**Figure 7.**
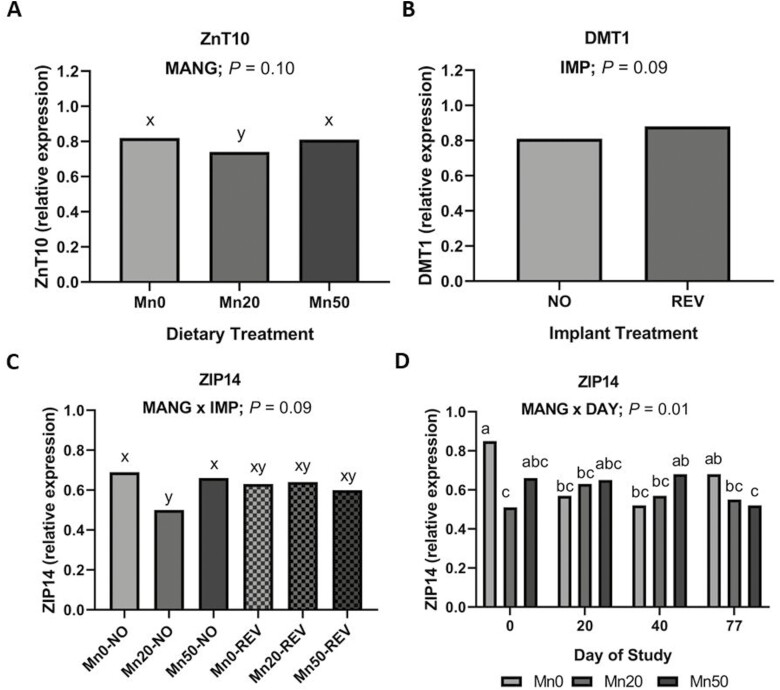
The effect of MANG on hepatic relative expression of ZnT10, effect of IMP on relative expression of DMT1, the effect of MANG × IMP and MANG × DAY on ZIP14 relative expression. Data were analyzed as repeated measures of the mixed procedure of SAS. Within a panel, ^a,b^unlike superscripts differ (*P* ≤ 0.05); ^x,y^unlike superscripts tend to differ (0.05 < *P* ≤ 0.10).

Relative expression of solute carrier family 39 member 8 (SLC39A8), which encodes the protein ZIP8, responsible for cytosolic influx of Zn and Mn was not influenced by IMP, MANG, and DAY or their interactions (*P *≥ 0.13). However, for solute carrier family 39 member 14 (SLC39A14), which encodes the protein ZIP14, a MANG × DAY effect was noted (Figure 7D; *P *= 0.01) where Mn0 had the greatest expression on day 0 with Mn 20 being lowest, and Mn50 intermediate. Expression of ZIP14 was similar on days 20 and 40. On day 77 Mn0 had greater ZIP14 expression compared to Mn50 with Mn 20 being intermediate. Additionally, a tendency for a MANG × IMP effect was noted (Figure 7C; *P *= 0.08) for ZIP14 driven by Mn20-NO having the lowest expression compared to Mn0-NO and Mn50-NO while all three implant treatments were intermediate.

Relative expression of solute carrier family 30 member 10 (SLC30A10), which encodes the protein Znt10, a Mn efflux transporter, tended to be lesser in Mn20 compared to Mn0 and Mn50 (Figure 7A; MANG; *P *= 0.10). Relative expression of another hepatic Mn importer, divalent metal-ion transporter 1 (DMT1) tended to be greater in cattle given a steroidal implant compared to NO (Figure 7B; IMP; *P *= 0.09).

#### Enzyme activity

**Figure 8.**
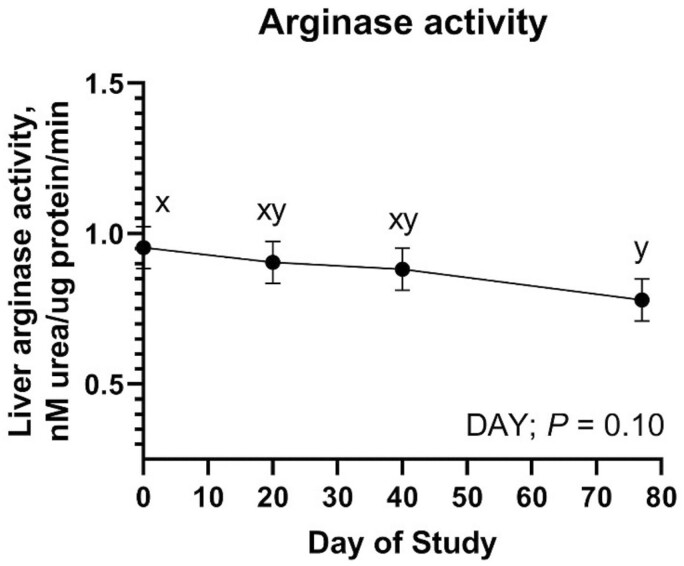
The effect of DAY on hepatic arginase activity. Data were analyzed as repeated measures of the mixed procedure of SAS. Within a panel, ^a,b^unlike superscripts differ (*P* ≤ 0.05); ^x,y^unlike superscripts tend to differ (0.05 < *P* ≤ 0.10).

Hepatic arginase activity was measured on days 0, 20, 40, and 77 relative to implant. Hepatic arginase activity was not influenced by MANG, IMP, MANG × DAY, IMP × DAY, or MANG × IMP (*P *≥ 0.15). Hepatic arginase activity did tend to decrease over time (Figure 8; DAY; *P *= 0.10).

**Figure 9.**
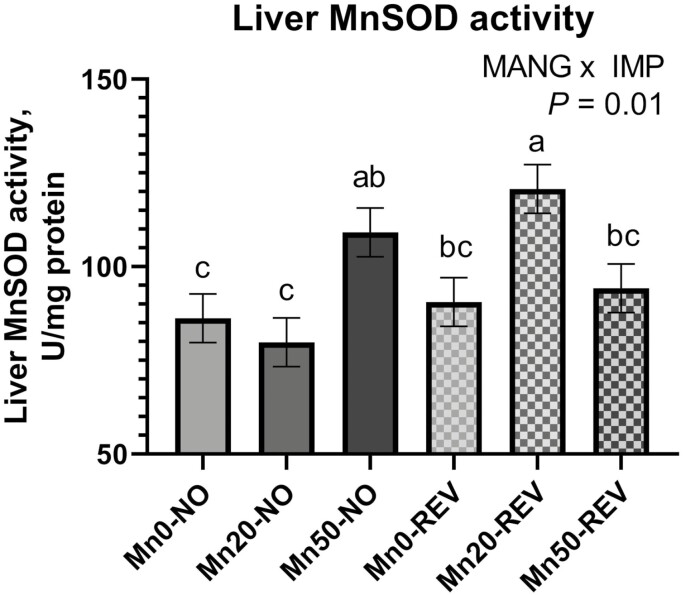
The effect of MANG × IMP on hepatic MnSOD activity. Data were analyzed as repeated measures of the mixed procedure of SAS. Within a panel, ^a,b^unlike superscripts differ (*P* ≤ 0.05); ^x,y^unlike superscripts tend to differ (0.05 < *P* ≤ 0.10).

Hepatic MnSOD activity was measured on days 0, 20, 40, and 77 relative to implant. Activity of hepatic MnSOD was not affected by MANG × IMP × DAY, MANG × DAY, or IMP × DAY (*P *≥ 0.54). Activity of hepatic MnSOD was greatest on day 0, lesser on days 20 and 40, and intermediate on day 77 (DAY; *P *= 0.02). Values for MnSOD activity were 105.7, 92.3, 90.2, and 99.0 (SEM = 4.41) U/mg/protein on sampling days 0, 20, 40, and 84, respectively. Additionally, a significant MANG × IMP effect (Figure 9; *P *= 0.01) was noted where Mn20-REV had the greatest MnSOD activity, followed by Mn50-NO, then Mn50-REV and Mn0-REV, and lowest was Mn0-No and Mn20-NO. Within the MANG × IMP effect, REV tended to have greater overall MnSOD activity compared to NO (IMP; *P* = 0.09).

## Discussion

Manganese is an essential trace element involved in numerous metabolic and physiological pathways related to growth. We have noted liver Mn consistently decreases following implant (Messersmith, 2018; Reichhardt et al., 2021; Messersmith et al., 2022), suggesting either increased demand for Mn or changes in liver Mn metabolism. Additionally, roles for Mn related to skeletal muscle growth have been reported (Gordon et al., 2019). This study aimed to determine how increasing supplemental Mn concentration influences steroidal implant-induced growth, carcass characteristics, TM status, circulating metabolites, hepatic expression of genes related to arginine metabolism, urea synthesis, antioxidant capacity, and TM homeostasis and activity of hepatic arginase and MnSOD, as well as markers of TM metabolism in implanted vs. non-implanted finishing beef steers.

Kemmerer et al. (1931) established the essentiality of Mn in mice and Mn deficiency in ruminants was evaluated for the first time by Bentley and Phillips (1951). A survey of feedlot consulting nutritionists (Samuelson et al., 2016) revealed Mn, in addition to other TM, is often supplemented in feedlot finishing diets at more than two times the current NASEM (2016) recommendation of 20 mg/kg DM. Niedermayer et al. (2018) noted industry supplementation rates resulted in a 13 kg advantage in HCW over un-supplemented cattle, and when steers were implanted, this was a 17 kg advantage. However, Niedermayer et al. (2018) supplemented several TM at increased concentrations concurrently and began dietary treatments at the time of implant. In the present study, Mn treatments began 55 d prior to implant administration and continued through the finishing phase. The basal concentration of 14 mg Mn/kg DM in the control diet was below NASEM (2016) recommendations but did not negatively affect cattle performance. While some interim performance effects of MANG were noted, overall, Mn supplementation did not increase cattle growth. Previous work reported similar results where increasing Mn supplementation to beef steers consuming a basal diet of just 8 mg Mn/kg DM (0 up to 240 mg Mn/kg of DM; Legleiter et al. [2005]) did not improve overall growth performance. However, MANG treatment did tend to influence RF accumulation where Mn50 had greater RF compared to Mn0 or Mn20. Connections between Mn and lipid metabolism have been reported previously using mice and rats as models (Baly et al., 1990). It was reported adipocytes from Mn-deficient rats had lesser glucose uptake and decreased triglyceride synthesis compared with rats fed adequate Mn (Baly et al., 1990). Additionally, greater dietary Mn has been shown to increase glucose uptake by rat adipocytes (Baquer et al., 2003). Legleiter et al. (2005) saw no difference in 12th RF, nor any other carcass characteristic. However, these workers reported muscle lipid content tended to respond in a quadratic fashion as dietary Mn increased. These data indicate the NASEM requirement is adequate and may be set as high as 20 mg Mn/kg DM to offset potential potent Mn antagonists such as Fe, common in cattle feedstuffs.

Unsurprisingly, steroidal implant administration increased SFBW, ADG, and G:F for all interim periods and on an overall basis (Johnson et al., 1996; Guiroy et al., 2002; Smith and Johnson, 2020a). Steroidal implant administration also increased HCW and tended to increase REA. Insulin sensitivity, based on the RQUICKI index value calculation (de Sousa et al., 2022) was assessed over the course of this study. Small differences existed between treatments resulting in a MANG × IMP for RQUICKI; however, perhaps most important to note is RQUICKI value decreased over time. Finishing cattle gain substantial amounts of empty body fat during the feeding period. Parallels have been drawn between human literature and beef cattle literature where fasting concentrations of insulin increase with obesity in humans or BW in beef steers (Eisemann et al., 1997; Smith et al., 2020b).

Cattle have very low intestinal absorption rates of dietary Mn at about 1% (Underwood, 1971; Sansom et al., 1978; Van Bruwaene et al., 1984). Homeostatic control of hepatic Mn is dynamic and fluctuates to provide adequate Mn status and avoid toxicity (Avila et al., 2013). Even in studies feeding excessive concentrations of supplemental Mn, hepatic Mn is very tightly controlled. Hansen et al. (2008) fed diets with dietary manganese (500 mg Mn/kg DM) at 25 times NASEM recommendations for 493 d. Hansen et al. (2008) found peak liver Mn was approximately 24 mg Mn/kg DM. In the present study, overall liver Mn concentration did increase with increasing supplemental Mn concentration. Legleiter et al. (2005) reported similar findings where a linear increase in liver Mn was observed with increasing dietary Mn. Liver Mn concentrations in that study ranged from 12.1 in controls to 15.1 mg/kg (DM basis) in steers supplemented with 240 mg of Mn-kg of DM (Legleiter et al., 2005). Thus, liver Mn lacks utility as a true biomarker of Mn status and may potentially be more responsive to physiological processes related to N metabolism. Similar to prior work (Messersmith, 2018; Reichhardt et al., 2021; Messersmith et al., 2022) liver Mn was lesser in implanted steers following implant administration, as we hypothesized. The prolonged decrease in liver Mn indicates a potential shift in hepatic Mn demand driven by changes brought on by implant administration.

To better understand the underlying mechanisms of this decrease in liver Mn, we aimed to quantify changes in gene expression of transporters involved in hepatic Mn homeostasis. Liver Mn homeostasis is facilitated by a group of trace metal transporters connecting circulation, hepatocytes, and the biliary system. Metal transporter ZIP14, plays a vital role in uptake of divalent metals into the liver and overall Mn homeostasis (Hennigar and McClung, 2016; Winslow et al., 2020), Znt10 controls hepatocyte Mn efflux to the bile to avoid excess Mn accumulation, and ZIP8 is responsible for cellular influx of Mn via reuptake from the biliary pool (Tuschl et al., 2012, 2016; Boycott et al., 2015). Thus, the biliary system of Mn serves as a reserve pool for Mn homeostasis. Our hypothesis was that both IMP and MANG would influence expression of this suite of Mn transporters.

Interestingly, relative expression of ZIP8 was not affected by MANG and neither ZIP8 nor ZnT10 were affected by IMP. Metal transporter ZIP8 has been shown to act through Mn to quantitatively modulate arginase activity (Lin et al., 2017) where ZIP8-KO mice had a significant decrease in hepatic arginase activity. However, given the lack of MANG and IMP effects on arginase activity reported herein, this is unsurprising. Transporter ZnT10, effectively a counterpart to ZIP8, controls hepatic Mn efflux. Individuals with ZnT10 mutations present with high concentrations of hepatic Mn (Tuschl et al., 2012). In this study, we found ZnT10 relative expression was greater in Mn50 and Mn0 compared to Mn20. Relative expression data for ZIP8 and ZnT10 do not fully support our initial hypothesis. Instead, the lack of difference between treatments suggests 14 to 64 mg total Mn/kg DM was physiologically similar in effects on hepatic Mn homeostasis. However, as a potential limitation of the PCR relative expression analysis, one must acknowledge the transient nature of mRNA and evidence indicating cellular abundance of proteins is primarily under the control of translation (Tian et al., 2004; Schwanhäusser et al., 2011). Thus, consensus between the transcriptome and proteome does not always exist (Dickson et al., 2007; Taquet et al., 2009).

Transporter ZIP14 mediates cellular influx of various trace metals from circulation, including Mn. The MANG × DAY effect for relative expression of ZIP14 was driven primarily by differences on days 0 and 77. Within the MANG × IMP effect the overall expression of ZIP14 in all three implanted treatments was similar. Relative expression of hepatic Mn importer, DMT1 tended to be greater in cattle given a steroidal implant compared to CON. From a homeostasis standpoint, upregulation of a Mn absorptive mechanism following the implant-induced decrease in liver Mn is expected. This is unusual as flexible hepatic excretion rate is the primary driver of Mn homeostasis (Britton and Cotzias, 1966; Miller, 1973; Hambidge et al., 1989) as 98% of absorbed Mn is excreted through the bile (Larsen et al., 1979; Hauser et al., 1994). Transporter DMT1 is not an exclusive transporter of Mn, and could be influenced by Fe or other divalent metals (Illing et al., 2012). As previously discussed, Hansen et al. (2008) reported excessive manganese diets (500 mg Mn/kg DM) caused suppression of DMT1. However, in the present study up to 50 mg supplemental Mn/kg DM did not influence relative expression of DMT1.

Both Mn (Horning et al., 2015) and steroidal implants (Lobley et al., 1985; Carmichael et al., 2018) have strong ties to whole-body N metabolism and protein synthesis. Steroidal implants decrease serum urea-N following implant administration (Bryant et al., 2010; Parr et al., 2014). As expected, we found implant administration decreased SUN relative to non-implanted steers. The increased net protein synthesis caused by steroidal implant administration may have simply prevented an increase in SUN in REV during this study. It is interesting to note the decrease in circulating SUN as a result of implant administration closely mirrors our consistently observed decrease in liver Mn. Thus, changes in hepatic and whole-body N metabolism may potentially be related to this change along with other Mn-dependent enzymes.

Arginase is a Mn-containing enzyme that mediates the hydrolysis of L-arginine to L-ornithine and urea in the final step of the urea cycle (Horning et al., 2015). More than 70% of cytosolic Mn is thought to be associated with arginase in the liver (Rosebrough et al., 1987). To investigate connections between decreased liver Mn and N metabolism we evaluated hepatic arginase activity and relative expression of ARG1 and ARG2. Recent work from our laboratory provides strong evidence the decrease in liver Mn may be related to arginase activity in hepatic tissue (Messersmith et al., 2022). Mechanistically, the hypothesis is implant administration decreases arginase activity due to increased net protein synthesis and decreased circulating SUN, thus resulting in lesser liver Mn concentration. Hepatic arginine metabolism is primarily balanced between arginase and nitric oxide synthase (Wu and Morris Jr, 1998). Interestingly IMP did not affect relative expression of ARG1 or ARG2. Arginase activity tended to decrease over time and contrary to our hypothesis, was also not significantly influenced by IMP or MANG. However, a MANG × DAY effect was noted for relative expression of ARG1, the cytosolic isoform of arginase more highly expressed in the liver (Caldwell et al., 2015), driven by increased relative expression of ARG1 in Mn20 on day 20. The MANG × IMP effect for relative expression of ARG1 seems to indicate that implant may drive changes in liver arginase and urea cycle, perhaps Mn in the diet also influences these factors. Relative expression of the mitochondrial isoform ARG2 changed over time but was not influenced by IMP or MANG. Though arginase competes with NOS2 for L-arginine (Wu and Morris Jr, 1998), no effects of MANG or IMP were noted on NOS2 expression or other genes related to urea synthesis. Our initial hypothesis was that implant administration would alter hepatic demand for enzymes related to urea synthesis due to increased net protein synthesis.

While a substantial proportion of Mn in the liver is likely associated with arginase, it also supports MnSOD. Manganese is important in mitigating mitochondrial oxidative stress via MnSOD (Miriyala et al., 2012). Concurrent with our relative expression data, implanting cattle tended to increase liver MnSOD activity. Expression and activity of MnSOD respond to oxidative stimuli (Esposito et al., 1999). Together, these results may be evidence of greater oxidative stress in implanted steers. The source of oxidative stress may potentially be related to increased overall hepatic metabolic demand due to anabolic stimulus. Literature has indicated Mn supplementation may influence MnSOD activity in human lymphocytes (Davis and Greger, 1992) but in this study, MANG did not affect overall MnSOD relative expression. Relative expression of NRF2, a regulator of cellular resistance to oxidants, was not influenced by IMP or MANG. Functionally, NRF2 helps control basal and induced expression of various genes to regulate the physiological and pathophysiological outcomes related to antioxidant capacity (Ma, 2013).

## Conclusions

This study represents the first investigation into increasing supplemental Mn and steroidal implant use in finishing beef cattle. Dietary Mn of 14 mg/kg DM did not seem to limit the growth of implanted or non-implanted cattle. This supports the current NASEM recommendation for dietary Mn where supplementing 20 mg Mn/kg DM may be sufficient to offset potential potent Mn antagonists such as Fe, common in cattle feedstuffs. Contrary to our hypothesis, arginase activity was not significantly affected by steroidal implant administration. Dietary Mn minimally influenced liver Mn concentration. In this study, it appears liver Mn concentration may be more responsive to stimuli, such as steroidal implants, that exert systemic influence resulting in altered hepatic Mn demand. Further investigation of how liver Mn concentration, serum urea-N concentration, and hepatic arginase activity are related may further elucidate how the systemic effects of steroidal implants influence liver Mn homeostasis. This study also found that MnSOD activity and relative gene expression are upregulated in implanted cattle, potentially due to increased metabolic load. Overall, this study provides an improved understanding of Mn supplementation and Mn homeostasis in the context of finishing beef cattle diets.

## Supplementary Material

skae062_suppl_Supplementary_Material

## Abbreviations

ADG: average daily gain
Akt: protein kinase B
BW: bodyweight
DMI: dry matter intake
DMT1: divalent metal transporter 1
E2: estradiol
EBF: empty body fat
HCW: hot carcass weight
ICP-OES: inductively coupled plasma optical emission spectroscopy
KPH: kidney, pelvic, and heart fat
Mn: manganese
MnSOD: Mn superoxide dismutase
mRNA: messenger ribonucleic acid
NEFA: non-esterified fatty acid
NOS: nitric oxide synthase
SUN: serum urea nitrogen
TBA: trenbolone acetate
TM: trace mineral
ZIP: Zrt/Irt-like proteins (SLC39 family)
Zn: Zinc
ZnT: Zn transporter (SLC30 family)
