# Supplementary material for: The influence of steroidal implants and manganese sulfate supplementation on growth performance, trace mineral status, hepatic gene expression, hepatic enzyme activity, and circulating metabolites in feedlot steers
Source: J Anim Sci. 2024 Mar 8;102:skae062. doi: 10.1093/jas/skae062 (PMC10959487; doi:10.1093/jas/skae062)
Supplement: skae062_suppl_Supplementary_Material [file skae062_suppl_supplementary_material.docx]

| **Supplemental Table S1.** Forward and reverse primers used for quantitative real-time PCR in hepatic tissue | | |
| --- | --- | --- |
| Name | Strand | Sequence (5'-3') |
| ARG1^1^ | Forward | TAATGGAAGTGAATCCGTCTC |
|  | Reverse | ACTTTGGTGGGCTAAGGTAAT |
| ARG2^2^ | Forward | TACGACCAACTTCCAACTCCC |
|  | Reverse | TCTGTCACCACAAACGCCTCA |
| CPS1^3^ | Forward | GCTAGCCTTAACACGACTGC |
|  | Reverse | TTCCCGTAGCCACTGTCCTA |
| DMT1^4^ | Forward | AGTTGCTCTGGGTTCTTCTGT |
|  | Reverse | CTGGGATACTGGCGGTGACAC |
| MnSOD^5^ | Forward | TGTGGGAGCATGCTTATTACCTT |
|  | Reverse | TGCAGTTACATTCTCCCAGTTGA |
| NFE2L2^6^ | Forward | CCCAGTCTTCACTGCTCCTC |
|  | Reverse | TCAGCCAGCTTGTCATTTTG |
| NOS2^7^ | Forward | CAGAAGGCCAAAGGGGATCT |
|  | Reverse | CCGGGGTCCTATGGTCAAAC |
| RPS9^8^ | Froward | CGCCTCGACCAAGAGCTGAAG |
|  | Reverse | CCTCCAGACCTCACGTTTGTTCC |
| SLC39A8^9^ | Forward | GGACTCAGCACCTCCATAGC |
|  | Reverse | GCCCACCAAGATGCCAAAAG |
| SLC39A14^10^ | Forward | GAGTTCCAGGAGTTCTGCCC |
|  | Reverse | ACGCAGAGGAGACCGTACC |
| SLC30A10^11^ | Forward | GCCCTGAATATCAGAGGGGT |
|  | Reverse | GCTGGGGTCAATGTAGCACT |
| ^1^Arginase 1, ^2^Arginase 2, ^3^Carbamoyl-phosphate synthase, ^4^Divalent metal transporter 1, ^5^Mn-dependent superoxide dismutase, ^6^Nuclear erythroid 2-related factor 2, ^7^Nitric oxide synthase 2, ^8^Ribosomal protein S9, ^9^Solute carrier family 39 member 8 (ZIP8), ^10^Solute carrier family 39 member 14 (ZIP14), ^11^Solute carrier family 30 member 10 (ZnT10) | | |

| **Supplemental Table S2.** Main effects of MANG and IMP on quantitative real-time PCR in hepatic tissue of beef steers. ^1^ | | | | | | | | | | |
| --- | --- | --- | --- | --- | --- | --- | --- | --- | --- | --- |
|  |  | MANG |  |  | IMP | |  |  | *P*-value |  |
|  | Mn0 | Mn20 | Mn50 |  | NO | REV |  | MANG | IMP | MANG*IMP |
|  |  |  |  |  |  |  |  |  |  |  |
| Gene |  |  |  |  |  |  |  |  |  |  |
| ARG1^2^ | 1.28 | 1.34 | 1.33 |  | 1.34 | 1.30 |  | 0.84 | 0.73 | 0.05 |
| ARG2^3^ | 0.93 | 0.84 | 0.91 |  | 0.86 | 0.93 |  | 0.67 | 0.45 | 0.22 |
| CPS1^4^ | 1.09 | 0.97 | 1.00 |  | 0.92 | 1.11 |  | 0.77 | 0.19 | 0.44 |
| DMT1^5^ | 0.85 | 0.81 | 0.87 |  | 0.81y | 0.88x |  | 0.35 | 0.09 | 0.57 |
| MnSOD^6^ | 0.98 | 0.94 | 0.97 |  | 0.91b | 1.03a |  | 0.76 | 0.03 | 0.09 |
| NFE2L2^7^ | 0.89 | 0.83 | 0.88 |  | 0.83 | 0.90 |  | 0.79 | 0.30 | 0.41 |
| NOS2^8^ | 1.03 | 1.01 | 1.04 |  | 1.08 | 0.98 |  | 0.97 | 0.29 | 0.97 |
| ZIP8^9^ | 0.81 | 0.74 | 0.77 |  | 0.75 | 0.80 |  | 0.54 | 0.26 | 0.23 |
| ZIP14^10^ | 0.66 | 0.57 | 0.63 |  | 0.61 | 0.62 |  | 0.21 | 0.79 | 0.09 |
| ZnT10^11^ | 0.82x | 0.74y | 0.81x |  | 0.78 | 0.80 |  | 0.10 | 0.46 | 0.24 |
| ^1^Data represent the main effects of MANG and IMP based on repeated measures analysis.  ^2^Arginase 1, ^3^Arginase 2, ^4^Carbamoyl-phosphate synthase, ^5^Divalent metal transporter 1, ^6^Mn-dependent superoxide dismutase, ^7^Nuclear erythroid 2-related factor 2, ^8^Nitric oxide synthase 2, ^9^Ribosomal protein S9, ^10^Solute carrier family 39 member 8 (ZIP8), ^11^Solute carrier family 39 member 14 (ZIP14), ^12^Solute carrier family 30 member 10 (ZnT10).  ^a,b^ Within rows, means without a common superscript differ (P ≤ 0.05).  ^x,y^ Within rows, means without a common superscript differ (0.05 < P ≤ 0.10). | | | | | | | | | | |

| **Supplemental Table S3.** Correlations between liver arginase activity, serum urea nitrogen, and liver manganese.^1^ | | | | |
| --- | --- | --- | --- | --- |
|  | Arginase activity^2^ | | SUN | |
|  | Corr^3^ | *P*-value | Corr^3^ | *P*-value |
|  | n = 72 |  | n = 72 |  |
| **Day 0** |  |  |  |  |
| SUN | 0.33 | 0.01 | - | - |
| Liver Mn | 0.35 | 0.01 | 0.20 | 0.12 |
| **Day 20** |  |  |  |  |
| SUN | 0.46 | 0.01 | - | - |
| Liver Mn | 0.51 | 0.01 | 0.60 | 0.01 |
| **Day 40** |  |  |  |  |
| SUN | 0.45 | 0.01 | - | - |
| Liver Mn | 0.37 | 0.01 | 0.29 | 0.02 |
| **Day 77** |  |  |  |  |
| SUN | 0.25 | 0.04 | - | - |
| Liver Mn | 0.07 | 0.61 | 0.20 | 0.11 |
| ^1^Serum for serum urea-N and liver samples for arginase activity and liver Mn concentration were collected on d 0, 20, 40, and 77 relative to implant administration.  ^2^Arginase activity measured as uM Urea/ug protein/min  ^3^Corr: r, Pearson’s correlation coefficient. | | | | |

| **Supplemental Table S4.** Correlations between liver arginase activity and relative expression of arginase 1 (ARG1) and arginase 2 (ARG2)^1^ | | | | |
| --- | --- | --- | --- | --- |
|  | Arginase activity^2^ | | ARG1 | |
|  | Corr^3^ | *P*-value | Corr^3^ | *P*-value |
|  | n = 48 |  | n = 48 |  |
| **Day 0** |  |  |  |  |
| ARG1 | -0.33 | 0.02 | - | - |
| ARG2 | 0.03 | 0.84 | 0.06 | 0.70 |
| **Day 20** |  |  |  |  |
| ARG1 | -0.35 | 0.01 | - | - |
| ARG2 | 0.06 | 0.70 | 0.22 | 0.14 |
| **Day 40** |  |  |  |  |
| ARG1 | -0.19 | 0.20 | - | - |
| ARG2 | -0.14 | 0.35 | -0.14 | 0.36 |
| **Day 77** |  |  |  |  |
| ARG1 | -0.26 | 0.07 | - | - |
| ARG2 | -0.10 | 0.50 | 0.33 | 0.02 |
| ^1^Liver samples for qPCR were collected on d 0, 20, 40, and 77 relative to implant administration.  ^2^Arginase activity measured as uM Urea/ug protein/min  ^3^Corr: r, Pearson’s correlation coefficient. | | | | |
